# Mucoadhesive Cationic Polypeptide Nanogel with Enhanced Penetration for Efficient Intravesical Chemotherapy of Bladder Cancer

**DOI:** 10.1002/advs.201800004

**Published:** 2018-03-27

**Authors:** Hui Guo, Faping Li, Weiguo Xu, Jinjin Chen, Yuchuan Hou, Chunxi Wang, Jianxun Ding, Xuesi Chen

**Affiliations:** ^1^ Department of Urinary Surgery the First Hospital of Jilin University Changchun 130021 P. R. China; ^2^ Key Laboratory of Polymer Ecomaterials Changchun Institute of Applied Chemistry Chinese Academy of Sciences Changchun 130022 P. R. China

**Keywords:** chemotherapy, mucoadhesion, orthotopic bladder carcinomas, penetrability, smart polypeptide nanogels

## Abstract

Initially, chemotherapy is effective for treatment of bladder cancer after transurethral resection of the bladder. However, certain patients progressively become unresponsive after multiple treatment cycles, which results from the rapid and almost complete excretion of clinically used formulations of antineoplastic agents with urinary voiding. Improving the mucoadhesiveness and penetrability of chemotherapeutic drugs are key factors in treatment of advanced bladder cancer. Here, a smart disulfide‐crosslinked polypeptide nanogel of poly(l‐lysine)–poly(l‐phenylalanine‐*co*‐l‐cystine) (PLL–P(LP‐*co*‐LC)) is developed to deliver 10‐hydroxycamptothecin (HCPT) for treatment of orthotopic bladder cancer. The positively charged PLL–P(LP‐*co*‐LC) can significantly prolong the retention period and enhance the tissue permeability of HCPT within the bladder wall of rat. Moreover, the reduction‐responsive polypeptide nanogel (i.e., NG/HCPT) possesses the capability to accurately and rapidly deliver HCPT in bladder cancer cells. NG/HCPT can significantly inhibit proliferation of human bladder cancer 5637 cells in vitro and enhance antitumor activity toward an orthotopic rat bladder cancer model in vivo. This work demonstrates that the smart polypeptide nanogel may function as a promising drug‐delivery system for local chemotherapy of bladder cancer with unprecedented clinical benefits.

## 1 Introduction

Bladder cancer (BC) is a heterogeneous disease with more than 75% of patients presenting with nonmuscle‐invasive bladder cancer (NMIBC) at first diagnosis.^1^ It is well known that BC preferentially afflicts men.^2^ The incidence rate is 3–4 times higher in men than in women.^3^ Nearly three‐fourths of patients with high‐grade NMIBC will experience tumor recurrence and progress within ten years.^4^ A strategy to reduce the high rate of recurrence and, potentially, progression of NMIBC is the use of intravesical instillation of chemotherapy after transurethral resection of the bladder.^5^, ^6^ Possible mechanisms are the eradication of floating tumor cells, an ablative effect on residual tumor cells at the tumor site, and an ablative effect on small overlooked tumors.^7^ However, the concentrations of the chemotherapeutic drugs decrease rapidly with the excretion of urine, resulting in a need for large dose and frequent drug instillation.^8^ It proves that the mucoadhesiveness and permeability of chemotherapeutic drugs will directly affect the drug absorption and chemotherapy efficacy.

To prolong the retention time and improve the permeability of chemotherapeutic drugs into the urothelium, mucoadhesive polymers have been widely developed as innovative nanoscopic drug delivery systems. These well‐designed drug carriers possess unique physical properties, including improved tissue mucoadhesiveness and permeability, colloidal stability, sustained drug delivery ability, as well as a relatively low cost.^9^, ^10^, ^11^, ^12^ However, there are still several deficiencies that limit the clinical applications. Take chitosan (CS) as an example, it has been extensively studied as a mucoadhesive natural polymer, which benefits from the spontaneous adhesion of the repeated cationic amino groups to the negatively charged mucin chains of mucosal surface.^13^ Thiolated CS nanoparticles showed a 170 times higher amount of remaining particles on the urinary bladder wall after 6 h compared with the fluorescent marker, fluorescein diacetate.^14^ However, it was reported that CS may cause deterioration and necrosis of the urothelium.^15^ More tolerable mucoadhesive materials are urgently needed to increase the exposure time of drugs in the bladder with a better ability of tissue penetration.

In our previous study, a reduction‐responsive cationic polypeptide nanogel (NG) poly(l‐lysine)–poly(l‐phenylalanine‐*co*‐l‐cystine) (PLL–P(LP‐*co*‐LC)) was synthesized to deliver 10‐hydroxycamptothecin (HCPT) for intravesical instillation of chemotherapy toward orthotopic mouse bladder cancer induced by *N*‐butyl‐*N*‐(4‐hydroxybutyl)‐nitrosamine (BBN) in drinking water.^16^ In order to improve the understanding of mucoadhesion and permeability of NG/HCPT, designed to enhance the targeted specific delivery, further investigation of the residence time and tissue penetrability of NG/HCPT within rat bladder wall was done in the present study. Considering ≈75% of BC cases are NMIBC at first diagnosis, 5637 cells, a high‐risk NMIBC cell line, was chosen for in vitro characterization.^17^ The orthotopic rat BC was chemically induced by *N*‐methyl‐*N*‐nitrosourea (MNU), which was close to human BC in the histopathology, biochemical properties, and biological behaviors.^18^ NG/HCPT designed to improve the curative effect and reduce normal tissue toxicity was systematically explored in vitro and in vivo. As mentioned above, CS was an extensively studied mucoadhesive natural polymer. A cationic nanoparticle (i.e., CS/HCPT) composed of CS and HCPT was prepared as a control. Encouragingly, the high urothelium adhesive property and penetration capacity, as well as the reduction‐responsive release ability enable NG/HCPT with extremely outstanding antitumor activity both in vitro and in vivo.

## 2 Results and Discussion

### 2.1 Intracellular Release and Cytotoxicity

**Figure 1:**
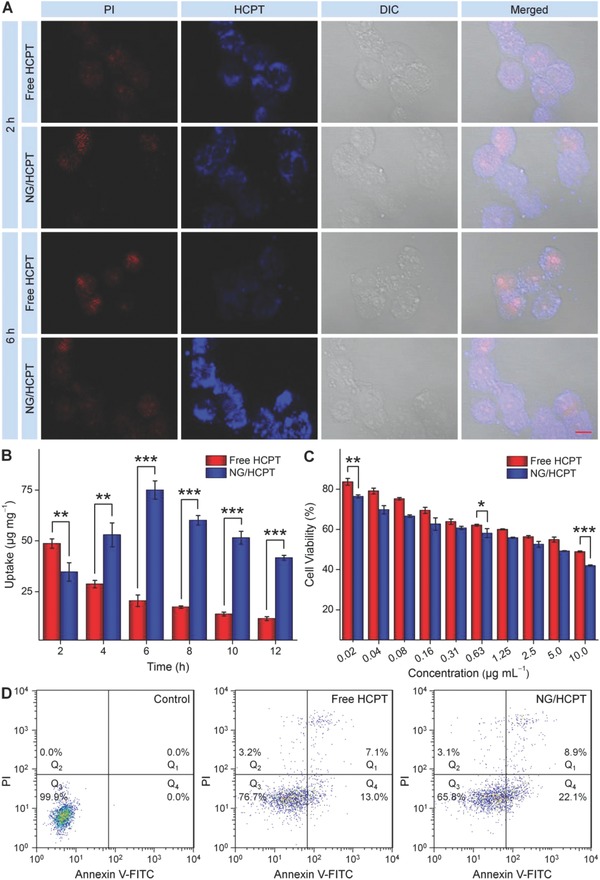
Cell uptake and cytotoxicity. A) Representative CLSM microimages of 5637 cells incubated with free HCPT or NG/HCPT for 2 and 6 h. For each panel, the microimages from left to right showed propidium iodide (PI, red), HCPT fluorescence in cells (blue), differential interference contrast (DIC) image, and the overlay of two images. The scale bar represents 10 µm. B) Internalization profiles of 5637 cells treated with free HCPT or NG/HCPT at a HCPT concentration of 1.25 µg mL^−1^. Data are presented as mean ± standard deviation (STD; *n* = 3; **P < 0.01, ***P < 0.001). C) In vitro cytotoxicities of free HCPT and NG/HCPT after incubation with 5637 cells for 24 h. Data are presented as mean ± STD (*n* = 3; *P < 0.05, **P < 0.01, ***P < 0.001). D) Apoptotic cell populations were determined by FCM analyses with Annexin V–fluorescein isothiocyanate (FITC) and PI staining after coincubating 5637 cells with PBS as a control, free HCPT, or NG/HCPT for 24 h. The lower‐left (Q3), lower‐right (Q4), upper‐right (Q1), and upper‐left (Q2) quadrants in each panel indicated the populations of normal, early apoptotic, late apoptotic, and necrotic cells, respectively.

Confocal laser scanning microscopy (CLSM) was performed to qualitatively assess the cell uptake and intracellular release behaviors of NG/HCPT. As shown in **Figure** **1**A, slightly higher HCPT fluorescence intensity was observed in the cells incubated with free HCPT as compared to that of NG/HCPT at 2 h. It was reported that free HCPT was transported into cytoplasm via simple diffusion, while the internalization of NG/HCPT was presumably through an endocytosis pathway.^19^, ^20^ The phenomenon demonstrated that free HCPT could enter into 5637 cells with a higher speed via diffusion at 2 h. With the incubation time extended to 6 h (Figure 1A), the HCPT fluorescence of cells treated with free HCPT became extremely weak. However, the cells treated with NG/HCPT depicted a significantly enhanced HCPT fluorescence. The high intracellular HCPT concentration in the cells treated with NG/HCPT benefited mainly from both the positive charge and the disulfide‐crosslinked property of PLL–P(LP‐*co*‐LC). The positively charged NG/HCPT was adhered to the negatively charged cell membrane by electrostatic interaction and internalized into the cytoplasm efficiently.^21^ Subsequently, the high glutathione (GSH) concentration in 5637 cells triggered cleavage of the disulfide bond and further resulted in the release of HCPT from NG/HCPT.^22^

The cell uptake and intracellular release behaviors were further confirmed quantitatively by microplate reader as described by Wei et al.^23^ At 2 h, the content of HCPT in the cells treated with free HCPT was a little higher than that of the cells treated with NG/HCPT. As the incubation time prolonged, the uptake of free HCPT was declined, while the uptake of NG/HCPT rapidly increased and reached its peak at 6 h, then decreased slowly. Conversely, after 6 h incubation, the content of HCPT in the cells treated with NG/HCPT was 3.7 times higher than that of the free HCPT‐treated one (Figure 1B), which was consistent with the results determined by CLSM (Figure 1A).

To assess potential cytotoxicity of NG/HCPT, the standard methyl thiazolyl tetrazolium (MTT) assay was carried out. BC 5637 cells were incubated with free HCPT or NG/HCPT at different concentrations for 24 h. Concentration‐dependent cytotoxicity of these different HCPT formulations was observed (Figure 1C). NG/HCPT exhibited higher cytotoxicity in comparison with free HCPT against 5637 cells. The better cell proliferation inhibition effect of NG/HCPT was probably attributed to the improved endocytosis, rapid intracellular HCPT release, and high HCPT concentration within 5637 cells. Importantly, the 5637 cells incubated with NG/HCPT displayed a lower half maximal inhibitory concentration (IC_50_) of 3.1 μg mL^−1^ than those treated with free HCPT (i.e., 9.8 μg mL^−1^). The IC_50_ value quantitatively confirmed a better cytotoxicity of NG/HCPT compared to free HCPT. The reduction‐responsive property and enhanced cytotoxic effect endow NG/HCPT with great potential for BC chemotherapy and made a better antitumor activity in vivo possible.

Simultaneously, MTT assay was performed with a noncancer cell line, i.e., NIH3T3 fibroblast cells, to establish the cytotoxicity and selectivity of NG/HCPT. As depicted in Figure S4 (Supporting Information), NG/HCPT showed higher cytotoxicity in comparison with free HCPT against NIH3T3 cells. The cytotoxicity of NG/HCPT against NIH3T3 cells was obviously lower than that of 5637 cells. The excellent selectivity of NG/HCPT was benefited from the different GSH concentrations between normal cells and BC cells. It was reported that tumor tissues showed higher concentration of GSH compared with normal tissues.^24^

To evaluate the apoptosis of BC cells induced by NG/HCPT, 5637 cells were exposed to free HCPT or NG/HCPT solution at an equivalent HCPT dosage of 0.2 μg mL^−1^ for 24 h. The cells were double stained for viability and apoptosis, and analyzed by flow cytometry (FCM) analysis. As shown in Figure 1D, the viability of 5637 cells was significantly reduced after treatment with different HCPT formulations. Free HCPT contributed to 13.0% early apoptotic cells and 7.1% late apoptotic cells. While for NG/HCPT, the level was up to 22.1% and 8.9%, respectively. The results were consistent with the MTT assay. This may be owing to the fact that NG/HCPT was internalized in 5637 cells effectively, trigged to release HCPT rapidly, and accumulated in the nuclei with a high level of the drug.

### 2.2 Mucoadhesiveness, Permeability, and Biodistribution

The mucoadhesiveness and permeability of NG/HCPT were confirmed by CLSM. The tumor‐bearing rats were anesthetized and then free HCPT or NG/HCPT solution at a equivalent HCPT dosage of 6.0 mg per kg body weight (mg (kg BW)^−1^) was intravesically instilled into the rat bladders through the urethra. At different predetermined time points (i.e., 0.5, 2, 6, 12, 24, and 48 h), the bladders were harvested and cut into bladder sections. The urothelial surface and full‐thickness bladder wall were observed by CLSM to confirm the mucoadhesiveness and permeability, respectively.

**Figure 2:**
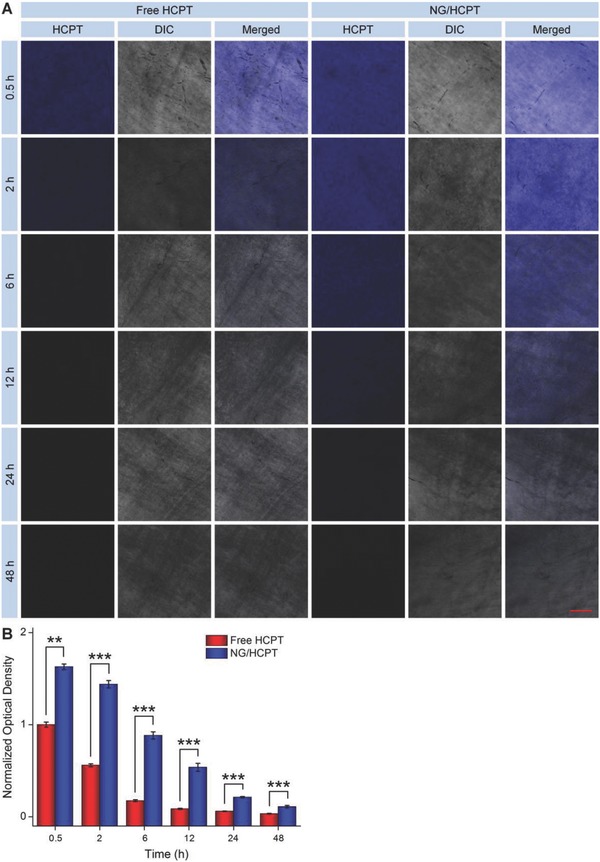
Mucoadhesiveness. A) Urothelial surface observed by CLSM to investigate the mucoadhesion of free HCPT and NG/HCPT. B) Statistical analysis of optical density of HCPT fluorescence intensity. The scale bar in (A) represents 100 µm. Data are presented as mean ± STD (*n* = 3; **P < 0.01, ***P < 0.001).

As depicted in **Figure** **2**A, at all the time points, there was an obviously stronger HCPT fluorescence in the bladders of NG/HCPT‐treated rats than that in the bladders of free HCPT‐treated rats. The strongest HCPT fluorescence was recorded in the bladder exposed to NG/HCPT for 0.5 h. The HCPT fluorescence intensity of bladders treated with free HCPT decreased rapidly, while the NG/HCPT‐treated bladders continually showed relatively high HCPT fluorescence intensity. This phenomenon indicated the excellent mucoadhesiveness of NG/HCPT. The mucoadhesiveness was further quantitated with ImageJ software (National Institutes of Health; Bethesda, MD, USA). The HCPT fluorescence intensity of bladder in the free HCPT group at 0.5 h was defined as “1”. The relative HCPT fluorescence intensity of bladders in the free HCPT group or NG/HCPT group was recorded as a ratio. As shown in Figure 2B, the signal of HCPT in the bladder of NG/HCPT group was 1.6 times higher than that in the bladder of free HCPT group at 0.5 h, 2.6 times higher at 2 h, and 5.1 times higher at 6 h. The quantitative data more convincingly confirmed the excellent mucoadhesiveness of NG/HCPT. As depicted in Figure S3 (Supporting Information), the HCPT fluorescence of bladder in the CS/HCPT group decreased slowly and maintained a relatively high level, but still significantly lower than that of NG/HCPT at all time intervals. This phenomenon indicated a good mucoadhesiveness of CS/HCPT with a sustained release property. However, the mucoadhesiveness of CS/HCPT was still much lower than that of NG/HCPT.

**Figure 3:**
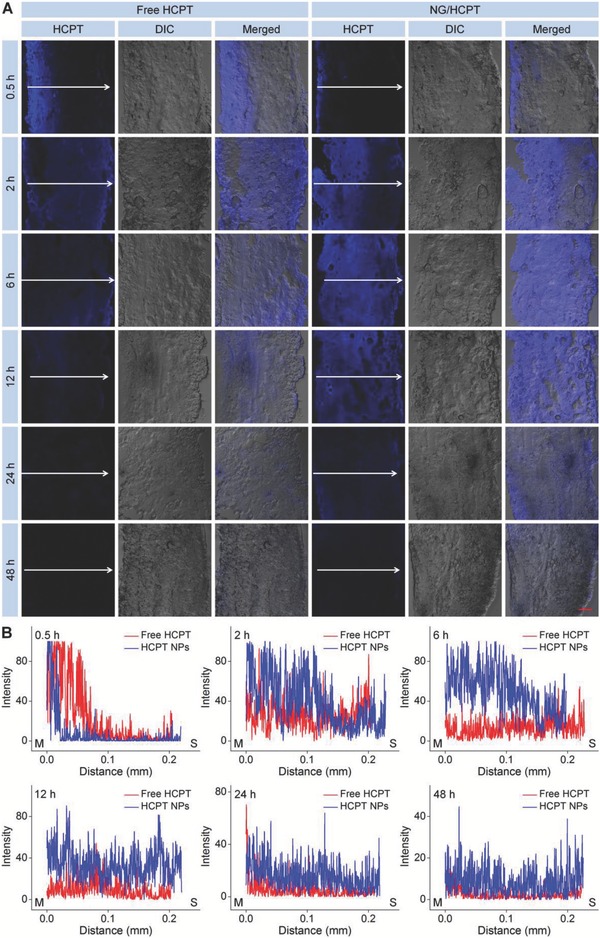
Penetrability. A) Penetrability of free HCPT and NG/HCPT investigated by CLSM. B) Statistical analysis of optical density of HCPT fluorescence intensity. The arrow represents the penetration direction of HCPT. The scale bar in (A) represents 100 µm. M, mucous membrane; S, serous membrane.

To determine the permeability of NG/HCPT, the penetration depth of HCPT formulations was detected and shown in **Figure** **3**A. At 0.5 h, the fluorescence signal was detected in almost the entire bladder wall, which was especially strong near the mucous membrane in the free HCPT group, while the HCPT fluorescence in the bladder of NG/HCPT group was relatively weak and confined to the mucous membrane. However, as time passed, NG/HCPT gradually penetrated through the whole bladder wall and displayed a relatively higher HCPT fluorescence compared to free HCPT, which was mainly due to the positive surface of NG/HCPT.^25^ This phenomenon indicated that the bladder exposure to NG/HCPT could maintain a high level of HCPT, which was vital for BC chemotherapy. To further confirm the permeability of NG/HCPT, the relative optical density of HCPT was quantified with ImageJ software. The optical density of maximum value in the bladder of free HCPT group at 0.5 h was defined as “100”. The relative optical density of free HCPT or NG/HCPT group was calculated as the ratio to maximum value, which was shown in Figure 3B. NG/HCPT could penetrate the bladder wall and maintain a higher concentration in bladder for a longer period as compared with free HCPT. The quantitative results predicted better therapeutic effect of NG/HCPT than that of free HCPT for BC. The HCPT fluorescence of the bladder samples treated with CS/HCPT was lower than that of free HCPT at first and then reversed (Figure S3, Supporting Information), whereas it was weaker than that of NG/HCPT during the detection. All these results suggest that CS/HCPT has a good penetrability and delivered the HCPT in a sustained release manner, while the permeability of CS/HCPT was still worse than that of NG/HCPT.

The in vivo biodistribution of NG/HCPT was conducted by high‐performance liquid chromatography (HPLC). At 6 h after intravesical instillation of free HCPT or NG/HCPT in male Sprague‐Dawly (SD) rats, the bladders and other major organs (i.e., the heart, liver, spleen, lung, and kidney) were harvested, homogenized, acidified, and followed by an extraction in acetonitrile/methanol (1/1, v/v). After centrifugation, 20.0 µL of clear suspension was determined by HPLC. As depicted in Figure S5 (Supporting Information), the bladder in the NG/HCPT group provided an extremely high HCPT content, which was 3.4 times higher than that of the free HCPT group. In addition, NG/HCPT was little accumulated in the other organs. The phenomenon could not only improve the therapeutic effect of HCPT, but also reduce the side effects. It was also an important predictor of better antitumor activity of NG/HCPT than that of free HCPT in vivo.

### 2.3 In Vivo Antitumor Efficacy

**Scheme 1:**
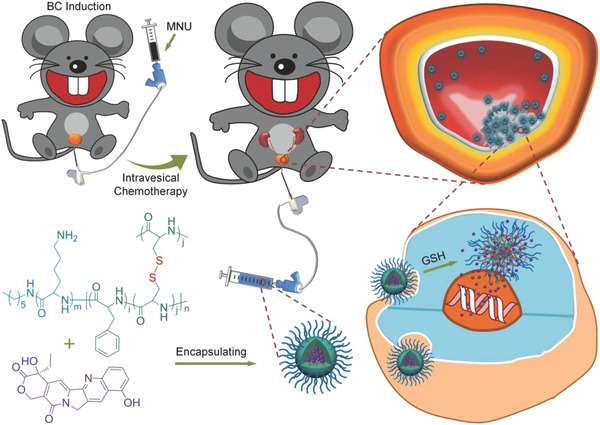
Schematic illustration of chemical structure of NG/HCPT and its metabolic process in vivo. The orthotopic rat BC was successfully induced by MNU. After intravesical instillation, NG/HCPT was selectively accumulated in tumor tissue because of the strong mucoadhesive properties and internalization by cancer cells. The high intracellular GSH concentration triggered the cleavage of the disulfide bond and further resulted in the selective release of HCPT from NG/HCPT.

To investigate the antitumor efficacy of NG/HCPT in vivo, orthotopic BC models in male SD rats were established. All animals received care in compliance with the guidelines outlined in the Guide for the Care and Use of Laboratory Animals, and all procedures were approved by the Animal Care and Use Committee of Jilin University. As depicted in **Scheme** **1**, the orthotopic BC was induced by intravesical instillation of MNU every other week for a total of eight weeks.^26^ To confirm whether the BC was successfully induced or not, at the end of the intravesical instillation of MNU, the histopathological examination was performed. As shown in Figure 5A, the cancer cells were larger with nuclear atypia and architectural disarray in comparison with the normal tissue cells, which indicated that the BC model was successfully established.

**Figure 4:**
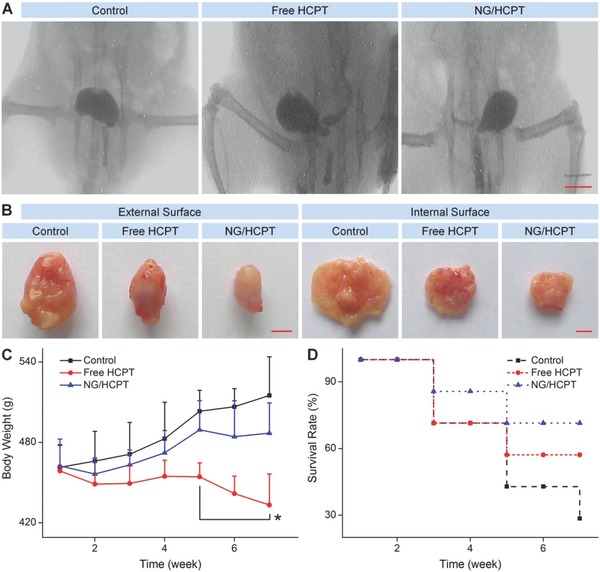
In vivo tumor inhibition. A) Cystography. B) Intact and opened bladders after the end of treatment. C) Body weight and D) survival rate during treatment with PBS as a control, free HCPT, or NG/HCPT. The scale bars represent (A) 1.0 cm and (B) 0.5 cm, respectively. Data are presented as mean ± STD (*n* = 7; *P < 0.05 refers to the statistically significant difference between the groups of free HCPT and NG/HCPT at each time point).

The rats with BC were randomly divided into three groups (*n* = 7 for each group) and treated with phosphate‐buffered saline (PBS), free HCPT, or NG/HCPT at an equivalent HCPT dosage of 6.0 mg (kg BW)^−1^ by intravesical instillation once a week for a total of six treatments. The cystography was monitored to define the tumor size and the progress of BC. Over the entire treatment period, the body weight was measured weekly as an indicator of systemic toxicity. At the end of treatment, the last cystography was given and shown in **Figure** **4**A. All the tumors showed a toothed margin or an irregular surface. The space‐occupying lesions of bladder in the PBS group were much larger than those of bladder in the free HCPT group, while no significant abnormalities were identified in the bladder of NG/HCPT group based on the smooth margin. According to the results, it was approved that NG/HCPT could dramatically inhibit the growth of BC as compared with free HCPT, which was most benefited from the remarkably improved mucoadhesiveness and permeability of NG/HCPT. The suboptimal antitumor activity of free HCPT was attributed to its quick excretion with urine.

The intact bladders were excised at the end of treatment and presented in Figure 4B. The outer surface of bladder in the PBS group was bumpy or dimpled, which was an appearance of the neoplasms infiltrating the muscular layer. However, the bladder treated with NG/HCPT displayed a smooth surface, a normal size, and a general shape, just like the healthy rat bladder. The bladder in the free HCPT group was somewhere in the middle. Then, the bladders were incised to provide a better understanding of the inner surface (Figure 4B). It could be seen that multiple neoplasms projected on the inner surface and grew into the cavity in the control and free HCPT groups, while no appreciable neoplasms were identified in the NG/HCPT group. The results confirmed the inhibition effect of NG/HCPT toward BC again.

In order to assess the toxicity of NG/HCPT in vivo, the change in body weight was monitored weekly. As shown in Figure 4C, during the entire process of chemotherapy, the rats treated with NG/HCPT did not show significant weight loss, suggesting a negligible toxicity of NG/HCPT. The survival rate is another necessary indicator of therapeutic effect and systemic toxicity.^27^ The survival periods of rats treated with different HCPT formulations were prolonged as compared with the control group. The rats treated with NG/HCPT survived somewhat longer than those treated with free HCPT (Figure 4D), which was consistent with the tumor inhibitory data. NG/HCPT showed the property of controlled release of HCPT and could selectively accumulate in the tumor site, which enhanced the tumor growth inhibition effect and decreased the side effects. All these results demonstrated that NG/HCPT had an advantage of biological safety.

**Figure 5:**
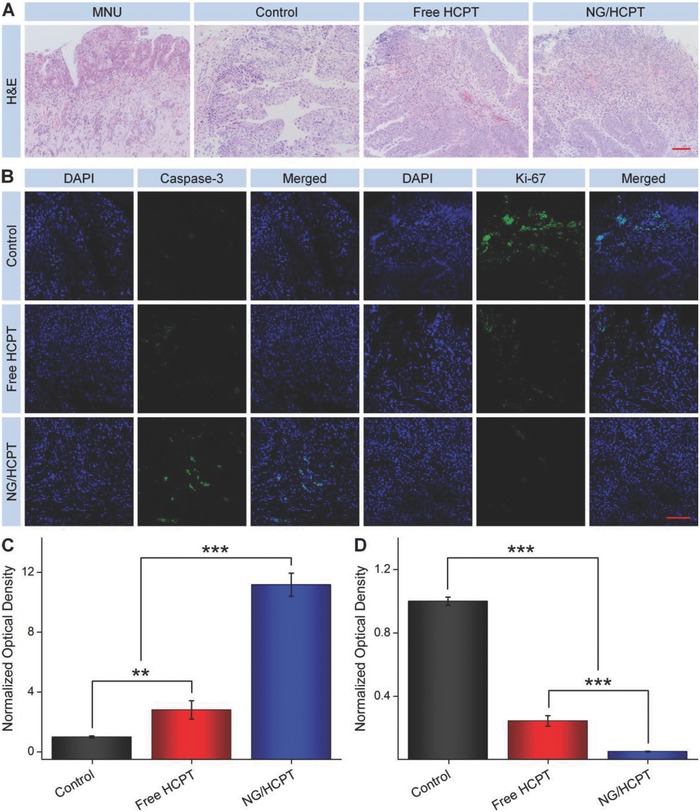
Histopathology and immunohistochemistry. A) Histopathological (i.e., H&E) and B) immunohistochemical (i.e., Caspase‐3 and Ki‐67) analyses of tumor tissue sections after treatment with PBS as a control, free HCPT, or NG/HCPT. The scale bars in (A) and (B) represent 200 and 100 µm, respectively. Relative optical densities of tumor sections from C) caspase‐3 and D) Ki‐67 after treatment with PBS as a control, free HCPT, or NG/HCPT. Data are presented as mean ± STD (*n* = 3; **P < 0.01, ***P < 0.001).

At the end of the intravesical instillation of different HCPT formulations, the bladders were excised from rats and sectioned for histopathological examination to further verify the antitumor efficacy of NG/HCPT. For hematoxylin and eosin (H&E) staining, the nuclei of cancer cells were spherical or spindle with obvious atypia compared to normal cells. However, the apoptotic cells had an ill‐defined morphology, and the nuclei became darker, pyknotic, and even disappeared. As shown in **Figure** **5**A, large amounts of tumor cells and few tumor necrosis areas were observed in the H&E‐stained tumor tissue section in the PBS group, indicating the active tumor growth. Fortunately, the nuclei started to become darker, pyknotic, and even disappeared in the bladder sections of both free HCPT and NG/HCPT groups. Distinguishable degrees of tumor necrosis were also observed. Among the three groups, the largest tumor necrosis areas were observed in the NG/HCPT group, indicating the significant antitumor activity of NG/HCPT. Furthermore, the tumor necrosis areas were calculated with NIS‐Elements imaging software (Nikon, NY, USA). The necrosis area of tumor section in the NG/HCPT group was (69.4 ± 3.1)%, which was 4.5 times higher than that of the free HCPT group (*P* < 0.01). Noteworthy, there was only (1.6 ± 0.3)% in the control group. The data of tumor necrosis area were in agreement with the in vivo antitumor efficiency.

Apoptosis includes a variety of apoptotic genes and caspase family is the crucial mediator of programed apoptosis. Among this family, caspase‐3 is an effect type of caspase and located on the downstream of apoptotic response.^28^ Ki‐67 is expressed from G1 phase to anaphase and thus provides a very good estimate of the fraction of a given cell population with cell‐cycle activity.^29^ The expressions of caspase‐3 and Ki‐67 were evaluated to further evaluate the apoptosis levels of cells by immunohistochemistry. As shown in Figure 5B, the most intense positive signal of caspase‐3 was observed in the NG/HCPT group as compared with the other two groups, which suggested that the amount of cells undergoing apoptosis was the largest in the NG/HCPT group. Moreover, immunohistochemical staining for Ki‐67 was performed to analyze the cell proliferation status, which was shown in Figure 5B. The largest number of Ki‐67 positive cells was observed in the PBS group, while the largest amount of Ki‐67 negative cells was occurred in the NG/HCPT group. The phenomenon indicated that BC cells grew the fastest in the PBS group and grew the slowest in the NG/HCPT group. All the results proved that NG/HCPT had a sustained HCPT release behavior to enhance tumor suppression and increase the apoptosis level in vivo. The relative optical densities of caspase‐3 and Ki‐67 were also calculated with similar mentioned embodiment as mucoadhesiveness.

The optical density of control group was defined as “1”, and the relative optical densities of free HCPT and NG/HCPT groups were calculated as a ratio. As shown in Figure 5C, the sorting of caspase‐3 amount was as follows: NG/HCPT > free HCPT > PBS as a control. Unexpectedly, the signal of NG/HCPT group was 3.9 times higher as compared with that of the free HCPT group, indicating the largest number of cell apoptosis in the NG/HCPT group. As shown in Figure 5D, the order of Ki‐67 amount was calculated as follows: PBS > free HCPT > NG/HCPT, indicating the weakest ability to proliferate in the NG/HCPT group. All these results confirmed the capability of NG/HCPT for BC chemotherapy.

## 3 Conclusion

In summary, a reduction‐responsive cationic NG/HCPT was developed for the potential intravesical chemotherapy of BC. The cationic PLL–P(LP‐*co*‐LC) promoted the mucoadhesion and penetrability of HCPT within the bladder wall. In addition, the reduction‐sensitive property gave NG/HCPT the ability to accurately release HCPT and specifically accumulate in the bladder cells when compared with free HCPT. Consequently, the intravesical instillation of NG/HCPT could significantly inhibit tumor growth in an orthotopic BC model. The significant proliferation inhibition in vitro and the promising orthotopic rat BC suppression in vivo demonstrate the great prospect of application of the smart polypeptide nanogel in intravesical chemotherapy.

## Conflict of Interest

The authors declare no conflict of interest.

## Supporting information

Supplementary
