# Supplementary material for: Mucoadhesive Cationic Polypeptide Nanogel with Enhanced Penetration for Efficient Intravesical Chemotherapy of Bladder Cancer
Source: Adv Sci (Weinh). 2018 Mar 27;5(6):1800004. doi: 10.1002/advs.201800004 (PMC6010003; doi:10.1002/advs.201800004)
Supplement: Supplementary file 1 — Supplementary [file ADVS-5-1800004-s001.pdf]

Copyright WILEY-VCH Verlag GmbH & Co. KGaA, 69469 Weinheim, Germany, 2018.

ADVANCED SCIENCE
Open Access

Supporting Information

for *Adv. Sci.,* DOI: 10.1002/advs.201800004

Mucoadhesive Cationic Polypeptide Nanogel with Enhanced Penetration for Efficient Intravesical Chemotherapy of Bladder Cancer

Hui Guo, Faping Li, Weiguo Xu, Jinjin Chen, Yuchuan Hou, Chunxi Wang, Jianxun Ding,* and Xuesi Chen*

Copyright WILEY-VCH Verlag GmbH & Co. KGaA, 69469 Weinheim, Germany, 2016.

Supporting Information

Mucoadhesive Cationic Polypeptide Nanogel with Enhanced Penetration for Efficient Intravesical Chemotherapy of Bladder Cancer

Hui Guo, Faping Li, Weiguo Xu, Jinjin Chen, Yuchuan Hou, Chunxi Wang, Jianxun Ding,* Xuesi Chen*

Dr. H. Guo, F. Li, Prof. Y. Hou, Prof. C. Wang
Department of Urinary Surgery, the First Hospital of Jilin University, Changchun 130021, P. R. China
E-mail: hou63@163.com
Dr. H. Guo, W. Xu, Dr. J. Chen, Prof. J. Ding, Prof. X. Chen
Key Laboratory of Polymer Ecomaterials, Changchun Institute of Applied Chemistry, Chinese Academy of Sciences, Changchun 130022, P. R. China
E-mail: jxding@ciac.ac.cn

Experiment Section

Materials: 10-Hydroxycamptothecin (HCPT) was purchased from Beijing Huafeng United Technology Co., Ltd. (Beijing, P. R. China). Chitosan (CS) was purchased from Zhejiang Golden Shell Pharmaceutical Co., Ltd. (Zhejiang, P. R. China). Clear polystyrene tissue culture treated 6-well and 96-well plates were obtained from Corning Costar Co. (Cambridge, MA, USA). Dulbecco's modified Eagle's medium (DMEM) and fetal bovine serum (FBS) were bought from Gibco (Grand Island, NY, USA). Methyl thiazolyl tetrazolium (MTT) and 4′,6-diamidino-2-phenylindole dihydrochloride (DAPI) were sourced from Sigma-Aldrich (Shanghai, P. R. China) and used as received. Propidium iodide (PI) was purchased from Beijing Dingguo Changsheng Biotechnology Co., Ltd. (Beijing, P. R. China). Acetic acid (HAc), ammonium acetate (NH_4Ac), and triethylamine (TEA) were purchased from Sinopharm Chemical Reagent Co., Ltd. (Shanghai, P. R. China). *N*-Methyl-*N*-nitrosourea (MNU) was purchased from Sichuan Hainuowei Technology Co., Ltd. (Sichuan, P. R. China).

22-Gauge closed IV catheter systems were purchased from Suzhou Bidi Medical Devices Co. Ltd. (Suzhou, P. R. China). All the other solvents and reagents were sourced from Sigma-Aldrich (Shanghai, P. R. China) and used as received. The purified deionized water was prepared by the Milli-Q plus system (Millipore Co., Billerica, MA, USA).

Preparation of HCPT-Loaded Cationic CS Nanoparticle (CS/HCPT): CS/HCPT was prepared through a facile nanoprecipitation method. In short, CS (50.0 mg) was dissolved in 20.0 mL of HAc solution and 25.0 mg of HCPT was dissolved in 20.0 mL of DMF by vortex and sonication. Then, 20.0 mL of HCPT solution was added to CS solution under stirring. The mixture was then stirred for 12 h at room temperature, followed by dialysis for 24 h. The dialysis medium was refreshed five times, and the whole procedure was performed in the dark. Then, the solution was filtered and lyophilized to obtain CS/HCPT.

Hydrodynamic Radii (R_hs) of CS/HCPT: R_hs were determined by dynamic light scattering (DLS) measurements on a WyattQELS instrument with a vertically polarized He−Ne laser (DAWN EOS, Wyatt Technology Co., Santa Barbara, CA, USA). The scattering angle was fixed at 90°. The samples were prepared in aqueous solution at a concentration of 100.0 μg mL^{-1}. Before measurements, the solution was filtered through a 0.45 μm Millipore filter.

Intracellular Drug Release: To evaluate the cell uptake and intracellular release behaviors of drug-loaded nanogel (*i.e.*, NG/HCPT), human bladder cancer (BC) 5637 cells were used. Confocal laser scanning microscopy (CLSM) was performed to qualitatively assess the cell uptake of NG/HCPT. 5637 cells were seeded onto glass coverslips in 6-well plates at a density of 1.5×10^5 cells in 2.0 mL of complete DMEM containing 10% (*V/V*) FBS, supplemented with 50.0 IU mL^{-1} penicillin and 50.0 IU mL^{-1} streptomycin per well, and cultured at 37 °C in 5% (*V/V*) carbon dioxide (CO_2) atmosphere for 24 h. Then the incubation medium was removed, and free HCPT or NG/HCPT in 2.0 mL of complete DMEM was added with a final HCPT concentration of 1.25 μg mL^{-1}. The cells incubated without NG/HCPT or free HCPT were used as a control. At predetermined time intervals, the culture

media were removed and the cells were washed three times with phosphate-buffered saline (PBS). Thereafter, the cells were fixed with 4% (*W/V*) PBS-buffered formaldehyde for 20 min at room temperature. Then, the cells were washed three times with PBS. 2.0 mL of 1% (*V/V*) Triton X-100 was dropped on the cells for membrane permeabilization. 12 min later, the cells were washed with PBS three times in the shaker. 2.0 mL 1.5 μg mL^{-1} PI was added to stain the cells for 15 min. The cells were then washed with PBS three times.[1] The microimages of cells were determined on a CLSM (LSM 780, Carl Zeiss, Jena, Germany). The cell uptake and intracellular release behaviors were further confirmed quantitatively by microplate reader as described by Wei *et al.*.[2] The cells were cultured in 96-well plates and then incubated with different HCPT formulations (1.25 μg mL^{-1}) for a desired period of time, then washed three times with ice-cold PBS. Cell lysis solution (1% (*V/V*) Triton X-100) was used to treat the samples, which was followed by the addition of sodium hydroxide (NaOH) to solute the internalized HCPT. After these treatments, the plate was measured at 384 nm by an Infinite M200 microplate spectrophotometer (Tecan, Durham, USA). Each experiment was performed in triplicate.

In Vitro Cell Proliferation Inhibition Assays: To assess the potential cytotoxicity of NG/HCPT, a standard MTT assay was carried out. The cells were planted into 96-well plates at 1.0×10^4 cells per well in 200.0 μL of complete DMEM and incubated at 37 °C for 24 h. Then the incubation medium was removed, and free HCPT or NG/HCPT in 200.0 μL of complete DMEM with various HCPT concentrations (0.02 – 10.0 μg mL^{-1}) was added. The cells without pretreatment were used as control. The cells were subjected to MTT assay after being incubated for another 24 h. The absorbance of the solution was measured at 490 nm on a Bio-Rad 680 microplate reader (Bio-Rad Laboratories, Hercules, CA, USA). The cell viability (%) was calculated according to Equation 1.

$$\text{Cell Viability (\%)} = \frac{A_{\text{sample}}}{A_{\text{control}}} \times 100\% \qquad (1)$$

In Equation (1), A_{sample} and $A_{control}$ represented the absorbances of sample and control wells, respectively.

Apoptosis Detections: The HCPT-induced apoptosis of 5637 cells was assessed by flow cytometry analyses (FCM). 5637 cells were seeded in 6-well plates at a density of 2×10^5 per well and incubated for 24 h. Subsequently, the incubation medium was removed and free HCPT or NG/HCPT in 2.0 mL of complete DMEM was added with a HCPT concentration of 0.2 μg mL^{-1}. The cells without pretreatment were used as a control. Cells were incubated for another 24 h at 37 °C and harvested with ethylenediaminetetraacetic acid (EDTA)-free trypsin, centrifuged at 3,000 rpm for 5 min and washed consecutively with PBS. Then the cells were resuspended in 0.5 mL binding buffer stained with 5.0 μL of Annexin V-FITC at room temperature for 10 min, followed by addition of 5.0 μL PI on ice. At the end of incubation, the cells were analyzed immediately by FCM. The first 10,000 events were acquired by CXP analysis software V2.1 (Applied Cytometry Systems, Dinnington, UK).

Animal Procedures: The male Sprague-Dawley (SD) rats weighing 170 – 200 g were purchased from the Laboratory Animal Center of Jilin University. All animals received care in compliance with the guidelines outlined in the Guide for the Care and Use of Laboratory Animals, and all procedures were approved by the Animal Care and Use Committee of Jilin University.

BC Model Induction and Histopathology Determinations: The orthotopic rat BC model was induced by intravesical instillation of MNU. Briefly, the rats were anesthetized with ether inhalation and then received 2.0 mg of MNU dissolved in 0.2 mL of sodium citrate buffer *via* a lubricated 22-gauge angiocatheter every other week for a total of eight weeks.[3] To avoid spontaneous micturition, the rats remained anesthetized for approximately 2 h after catheterization. The rats were observed once a day, weighed weekly, and palpated for bladder lesions every other day. At the end of MNU treatment, two rats were sacrificed randomly, and the bladders and major organs (*i.e.*, the heart, liver, spleen, lung, and kidney) were harvested,

fixed in 4% (*W/V*) PBS-buffered paraformaldehyde, and followed by dehydration, clearing, wax infiltration, and embedding. The sections of these embedded tissue samples were stained with hematoxylin and eosin (H&E) to assess the incidence and progressions of BC by microscope (Nikon TE2000U, Kanagawa, Japan).

Mucoadhesiveness and Penetrability Study: The mucoadhesiveness and penetrability of NG/HCPT were investigated by CLSM. First, the male SD rats with BC were anesthetized by an intraperitoneal injection of 10% (*W/V*) chloral hydrate. Then, the animals received an intravesical instillation of free HCPT or NG/HCPT solution with an equivalent HCPT concentration of 6.0 mg per kg body weight (mg (kg BW)$^{-1}$) *via* a lubricated 22-gauge closed IV catheters system. The rats were anesthetized for approximately 2 h and monitored carefully after catheterization. At predetermined time intervals, the bladders were excised, opened, and washed five times with PBS. For mucoadhesiveness study, tissue sections were prepared by cutting flattened bladder into square and washed thoroughly with PBS again. The urothelial surfaces of these prepared bladder sections were determined by CLSM immediately. For the penetrability study, the flattened bladders were embedded in Tissue-Tek O.C.T. Compound embedding medium (Miles Inc., Diagnostics Division, Elkhart, IN, USA). Then cryogenic sections of 6 μm thickness were sliced from mucous membrane to the serous membrane serially using a freezing microtome (Leica CM 1900, Wetzlar, Germany). The sections were then observed by CLSM.

In Vivo Biodistribution: The *in vivo* biodistribution of NG/HCPT was conducted by high-performance liquid chromatography (HPLC). Male SD rats with BC established as described above were treated with free HCPT or NG/HCPT at an equivalent HCPT dose of 6.0 mg (kg BW)$^{-1}$ by intravesical instillation, respectively (n = 3 for each group) and sacrificed 6 h later. The bladder and tested organs (*i.e.*, the heart, liver, spleen, lung, and kidney) were excised, washed, and accurately weighted. The tissue samples were homogenized with normal saline, which was acidified to pH 3.0 with HAc. Afterward, the tissue homogenates were extracted

with two volumes of cold mixture of acetonitrile/methanol (1/1, *V/V*). The clear suspensions were obtained by centrifugation at 15,000 rpm at 4 °C for 10 min and 20 μL of that was used to determine the concentration of HCPT by HPLC. Mobile phase was a mixture of acetonitrile/aqueous buffer (67/33, *V/V*), in which the aqueous buffer contained 0.5% (*V/V*) HAc, 75.0 mmol L^{-1} NH_4Ac, and 5.0 mmol L^{-1} TEA.[4,5]

In Vivo Antitumor Efficiency: A total of 21 male SD rats with BC were randomly divided into three groups, each consisted of seven rats. The animals were treated with PBS, free HCPT and NG/HCPT at an equivalent HCPT dose of 6.0 mg (kg BW)$^{-1}$ by intravesical instillation weekly for a total of six treatments. Cystography was used once a week for monitoring the tumor size and the progress of BC. The body weight was measured at the same time as an indicator of systemic toxicity.

Histological and Immunohistochemical Analyses: One week after the last intravesical instillation of various HCPT formulations, the rats were sacrificed and bladders were excised, fixed in 4% (*W/V*) PBS-buffered paraformaldehyde, and followed by dehydration, clearing, wax infiltration, and embedding. The sections of these embedded tissue samples were used for H&E staining. Some other sections were stained with the immunohistochemical method to assess the expression of caspase-3 (Abcam, Cambridge, MA, USA) and Ki-67 (Abcam, Cambridge, MA, USA). The alterations of histology were evaluated by microscope. The immunohistochemical staining results were examined by CLSM.

Statistical Analyses: All experiments were performed at least three times. The results were presented as means ± standard deviation (STD). Data were analyzed for statistical significance using Student's *t*-test. $^*P < 0.05$ was considered statistically significant, and $^{**}P < 0.01$ and $^{***}P < 0.001$ were considered highly significant.

Results and Discussion

Characterizations of CS/HCPT: HCPT was loaded into the core of CS through diffusion in a normal physiological aqueous environment. The drug loading content (DLC) of CS/HCPT was at a high level of 36.9 wt.%. The R_h of CS/HCPT detected by DLS was 59.2 ± 2.4 nm (Figure S1).

Cytotoxicity of CS/HCPT: The *in vitro* cytotoxicity of free HCPT and CS/HCPT against 5637 cells was estimated by a standard MTT assay. As shown in Figure S2, the cells treated with free HCPT or CS/HCPT exhibited cell proliferation inhibition efficacies in a concentration-dependent manner when compared with the control. At any given equivalent concentration, CS/HCPT induced more obvious inhibition in comparison with free HCPT toward 5637 cells. The profound cell killing effect of CS/HCPT was probably due to the spontaneous adhesion between CS/HCPT and 5637 cells, the improved endocytosis, as well as the deterioration and necrosis of cells induced by CS, which was mentioned in the "Introduction" part.[6]

Mucoadhesiveness and Permeability of CS/HCPT: The mucoadhesiveness and penetrability of CS/HCPT were investigated by CLSM. The rats were anesthetized and received an intravesical instillation of CS/HCPT solution through the urethra. At predetermined time intervals, the bladders were excised and prepared as bladder sections. For the mucoadhesiveness study, the urothelial surface was observed, and for the penetrability study, the full-thickness bladder wall was observed by CLSM.

As shown in Figure S3, there was a markedly lower HCPT fluorescence intensity in the bladder wall treated with CS/HCPT than that of free HCPT at first few time intervals, especially at 0.5 and 2 h. The HCPT fluorescence of CS/HCPT group decreased slowly and maintained a relatively high HCPT level. However, the HCPT fluorescence of the bladder samples treated with NG/HCPT was significantly higher than that of CS/HCPT at any time intervals (Figure 2). This phenomenon indicated a good mucoadhesiveness of CS/HCPT with a sustained release property. However, the mucoadhesiveness of CS/HCPT was still much

lower than that of NG/HCPT. For the penetrability study, the HCPT fluorescence of the bladder samples treated with CS/HCPT was lower than that of free HCPT at first and then reversed. During the detection, the HCPT fluorescence of CS/HCPT group was weaker than that of NG/HCPT group. All these results suggested that CS/HCPT possessed a good penetrability and delivered the HCPT in a sustained release manner, while the permeability of CS/HCPT was still worse than that of NG/HCPT.

[1] S. Zheng, C. Huang, X. Zhao, Y. Zhang, S. Liu, Q. Zhu, *Spectrochim. Acta, Part A* **2018**, *189*, 231.

[2] W. Wei, Z. G. Yue, J. B. Qu, H. Yue, Z. G. Su, G. H. Ma, *Nanomedicine* **2010**, *5*, 589.

[3] Q. Pan, G. L. Yang, J. H. Yang, S. L. Lin, N. Liu, S. S. Liu, M. Y. Liu, L. H. Zhang, Y. R. Huang, R. L. Shen, Q. Liu, J. X. Gao, J. J. Bo, *J. Exp. Clin. Cancer Res.* **2015**, *34*, 77.

[4] L. Zhang, M. Yang, Q. Wang, Y. Li, R. Guo, X. Jiang, C. Yang, B. Liu, *J. Controlled Release* **2007**, *119*, 153.

[5] P. Li, Y. Zheng, H. Ran, J. Tan, Y. Lin, Q. Zhang, J. Ren, Z. Wang, *J. Controlled Release* **2012**, *162*, 349.

[6] M. K. Kos, M. Bogataj, P. Veranic, A. Mrhar, *Biol. Pharm. Bull.* **2006**, *29*, 1685.

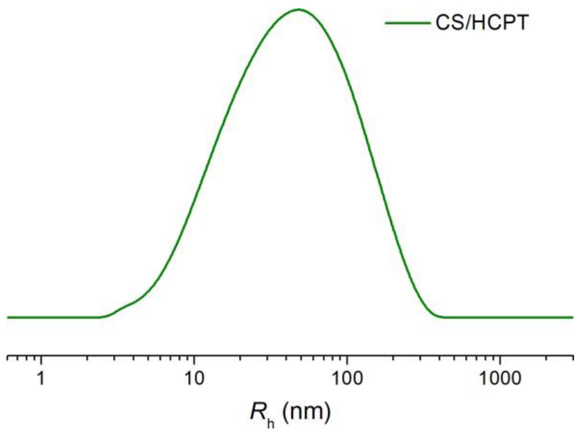

Figure S1. R_h of CS/HCPT.

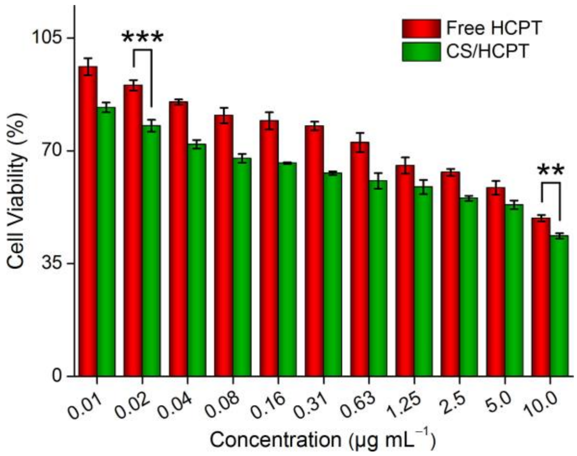

Figure S2. *In vitro* cytotoxicities of free HCPT and CS/HCPT after incubation with 5637 cells for 24 h. Data are presented as mean ± STD (n = 3; $**P < 0.01$, $***P < 0.001$).

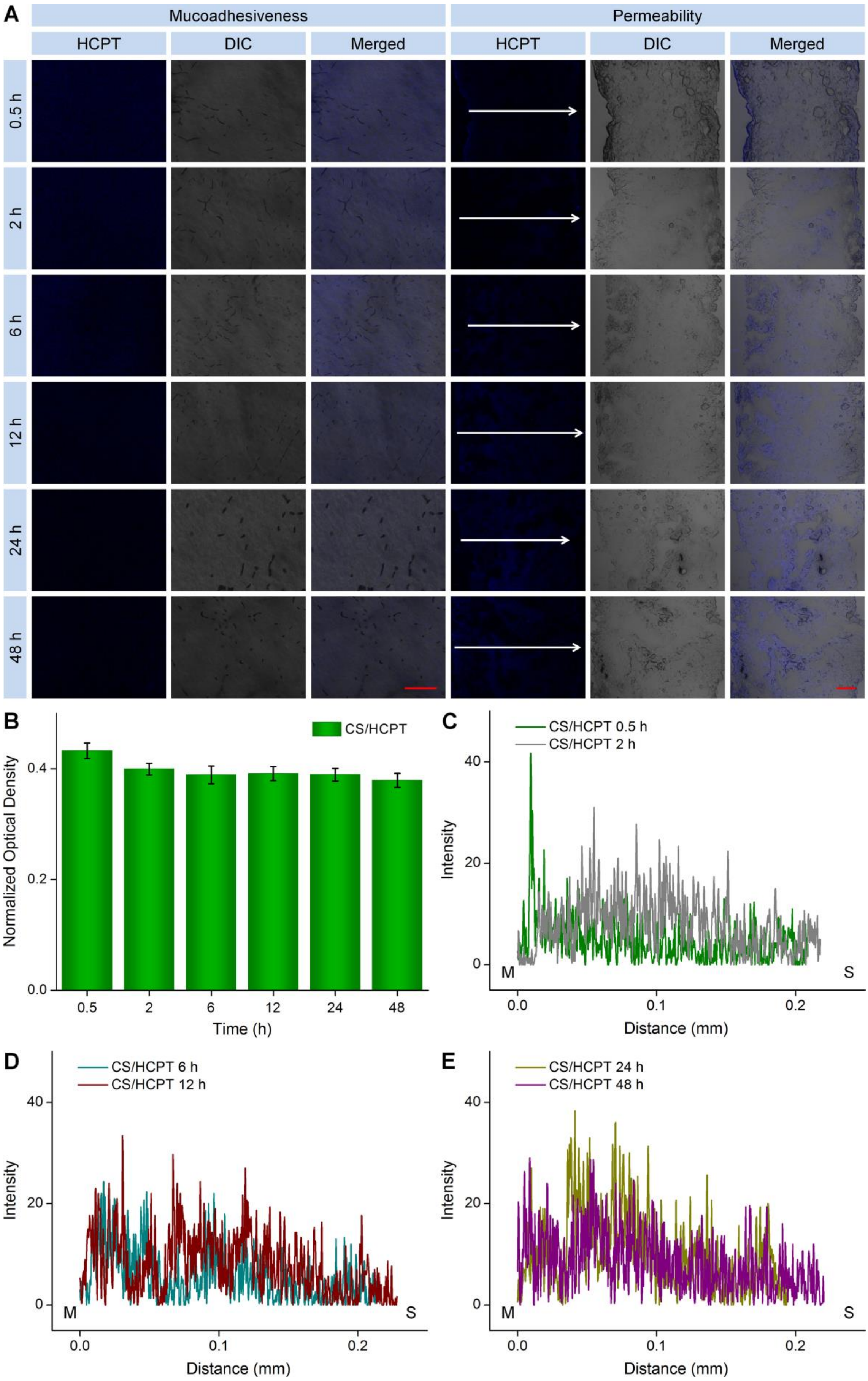
A
Mucoadhesiveness
Permeability
HCPT
DIC
Merged
HCPT
DIC
Merged
0.5 h
2 h
6 h
12 h
24 h
48 h
B
CS/HCPT
Normalized Optical Density
Time (h)
C
CS/HCPT 0.5 h
CS/HCPT 2 h
Intensity
Distance (mm)
M
S
D
CS/HCPT 6 h
CS/HCPT 12 h
Intensity
Distance (mm)
E
CS/HCPT 24 h
CS/HCPT 48 h
Intensity
Distance (mm)

Figure S3. Mucoadhesion and penetrability. (A) Mucoadhesion and penetrability of CS/HCPT investigated by CLSM. The scale bar represents 100 μm. (B) Bladder mucoadhesion quantification of CS/HCPT. (C, D, E) Bladder penetration quantification of CS/HCPT at different predetermined time points. The arrow represents the penetration direction of HCPT. M, mucous membrane; S, serous membrane.

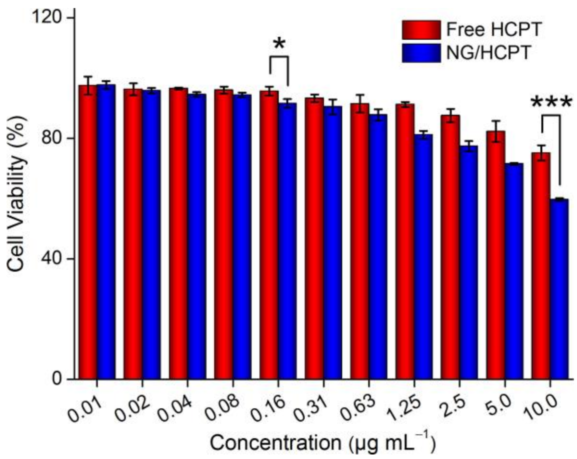

Figure S4. *In vitro* cytotoxicity of free HCPT and NG/HCPT after incubation with NIH3T3 fibroblast cells for 24 h. Data are presented as mean ± STD ($n = 3$; $^{*}P < 0.05$, $^{***}P < 0.001$).

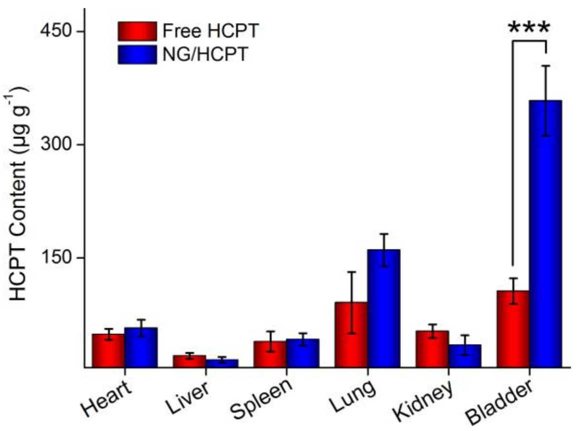

Figure S5. *In vivo* biodistribution of free HCPT and NG/HCPT. Data are presented as mean ± STD (n = 3; ***P < 0.001).
